# *In vitro* antioxidant and collagen restoration activities of a standardized *Centella asiatica* L. extract formulated with phospholipids

**DOI:** 10.3389/fmolb.2026.1832045

**Published:** 2026-06-29

**Authors:** Paola Nezi, Enrica Vella, Antonella Riva, Mattia Cicogni, Vittoria Cicaloni, Laura Salvini, Laura Tinti

**Affiliations:** 1 Botanicals Lab, Fondazione Toscana Life Sciences, Siena, Italy; 2 Product Portfolio Office, Indena SpA, Milan, Italy

**Keywords:** botanicals, *Centella asiatica*, collagen restoration, oxidative stress, phytosome

## Abstract

Skin aging is driven by the progressive accumulation of oxidative stress and chronic inflammation, leading to fibroblast dysfunction, extracellular matrix degradation, and impaired tissue repair. *Centella asiatica* L. is a botanical source rich in triterpenes with documented antioxidant, anti-inflammatory, and collagen-modulating activities; however, its biological efficacy is often limited by poor solubility and bioavailability. The present study aimed to evaluate the effects of a standardized *Centella asiatica* extract formulated with phospholipids (Centextra™) compared with an unformulated dry extract on oxidative stress, inflammatory stimuli and collagen homeostasis in an *in vitro* model of human dermal fibroblasts. Both products reduced intracellular ROS levels in a dose-dependent manner; however, the phospholipid-formulated extract exhibited significantly greater antioxidant efficacy, decreasing ROS levels to 115%–174% compared with 368% in cells treated with pro-oxidant stimulus (p < 0.0001). Centextra™ also significantly reduced IL-6 (∼75% vs. ∼150% in stimulated cells; p < 0.05) and MMP-1 levels (∼155% vs. higher induction in stimulated cells; p < 0.05). Furthermore, Centextra™ restored collagen levels to 85% vs. 73% impaired by inflammation (p < 0.05) and a greater migratory capacity, whereas the unformulated extract showed limited or no activity across these functional assays. Overall, the phospholipid formulation was associated with enhanced biological activity in this *in vitro* model, potentially reflecting improved cellular interaction or delivery of bioactive compounds. These findings provide a preliminary scientific rationale for the role of phospholipid-formulated *C. asiatica* in modulating skin-related contexts. Further studies are required to clarify the underlying mechanisms and confirm these findings in more physiologically relevant models.

## 1 Introduction

Aging is an unavoidable and universal biological process affecting all living organisms, driven by a combination of genetic, epigenetic, environmental influences that collectively shape the aging phenotype. Over time, humans experience the progressive accumulation of molecular, cellular, and tissue-level damage, which results in a gradual deterioration of physiological functions and a diminished capacity to maintain homeostasis (Ristori et al., 2025). Among different hallmarks involved in this physiological aging process, cellular senescence is one of the main steps that can be accelerated by oxidative stress and chronic inflammation (López-Otín et al., 2023). These damages can be easily detectable by skin texture, including increase of Transepidermal Water Loss (TEWL), skin cohesivity, skin firmness, decrease of skin elasticity and density. Skin represents the body’s largest and most visible organ, forming a complex and multifunctional barrier that protects the organism from environmental and physiological insults. It plays key roles in thermoregulation, sensory perception, immune defence, and protection against pathogens and chemical agents (Chin, et al., 2023). However, the skin is constantly challenged by both intrinsic factors, such as aging processes, Advanced Glycation End-products (AGE) formation, hormonal changes, and extrinsic factors, including ultraviolet (UV) exposure, air pollution, and reactive oxygen species (ROS). Together, these stressors accelerate oxidative damage, inflammation, and extracellular matrix (ECM) degradation, leading to structural and functional decline (Cruciani et al., 2025). Among dermal cell types, fibroblasts have a finite lifespan in cultures and are widely used as a model system for cellular aging (Kaji et al., 2009), for being essential for maintaining ECM homeostasis through the synthesis of collagen and elastin fibers, which ensure skin firmness and elasticity. Their impaired function is one of the hallmarks of skin aging, resulting in loss of structural integrity, delayed wound healing, and increased susceptibility to environmental stress (El-Sherbeni and Negm, 2023). Thus, strategies aimed at restoring fibroblast activity and reducing oxidative and inflammatory damage are of great interest in dermatological and cosmetic research. In recent decades, botanicals, phytochemicals, and nutraceuticals have attracted growing attention as potential modulators of cellular responses involved in skin aging. These natural compounds—often rich in polyphenols, flavonoids, and terpenes—exert antioxidant and anti-inflammatory effects, promote collagen synthesis, and support tissue repair (Ilma et al., 2025; Cruciani et al., 2025; El-Sherbeni and Negm, 2023). Their use in both topical and nutraceutical formulations align with the increasing demand for natural, sustainable, and effective bioactives. *Centella asiatica* L. is one of main botanical source known for its action in modulating the synthesis of collagen, and for its direct activity on fibroblasts during the scarring process (Maquart et al., 1999). *Centella asiatica L.*, also known as “gotu kola”, is a perennial, creeping herbaceous plant belonging to the *Apiaceae* (Umbelliferae) family, widely used in Asia, above all in Indian ayurvedic medicine, and in traditional African medicine. Centella grows easily in open warm, low and wet areas. Thanks to high quality phytochemical profile, due to the content of total triterpenes as hallmark bioactives (Hein et al., 2025)- divided into glycosides (madecassoside and asiaticoside) and aglycones (Asiatic acid and madecassic acid), it is used in traditional medicine, for the management of several conditions including as leprosy, varicose ulcers, eczema, diarrhea, fever and respiratory infections. Especially, aglycones are mostly documented to be able to simulate and improve wound repair and protect skin from photoaging (Dang et al., 2024; Ilma et al., 2025; Sucharitakul et. al, 2026).

In the Western medicine, Centella, mainly used as an extract, is employed thanks to its wide range of health benefits, particularly on tissue repair and microcirculation. In particular, Centella is endowed by a strong antioxidant power, effective on several forms of oxidative stress associated to inflammation or infections (Giacomelli et al., 2018).

However, an intrinsic problem associated with many botanical extracts, also the ones based on *Centella asiatica L.*, is their limited water solubility and, subsequently, low bioavailability, which often limits their application as a pharmacological agent. For this reason, a comparative study of a standardized phospholipid carrier-based (Phytosome™) *Centella asiatica* L. extract (Centextra™) vs. an unformulated dry *Centella asiatica L* (without a carrier system) was performed in order to investigate its antioxidative stress properties and its efficacy to restore collagen matrix under stress in a cellular model of skin. Based on the promising and consolidated data obtained in previous published studies conducted with other compounds and botanical extracts developed with Phytosome™ (Khan et al., 2026; Riva et al., 2016; Riva et al., 2019; Petrangolini et al., 2021; Cuomo et al., 2011), the same delivery system was applied to the *Centella asiatica L.* extract in order to obtain a stable and, food-grade formulation. The Phytosome™ technology constitutes a solid dispersion of botanicals or natural compounds into a 100% food-grade matrix, based on sunflower lecithin (phospholipids) amphipathic molecules, which act as inhibitor of self-aggregation and effective wetting agents, that also may permit a better cellular permeability. Thanks to the improved dissolution profile, enhanced by using a carrier, can let to consider this technology as a third-generation solid dispersion (Malkawi et al., 2022).

The Phytosome™ system contains the active ingredients of the botanical extract embedded in phospholipids. Thanks to the surfactant properties of phospholipids, this delivery system optimizes the dispersion in gastrointestinal environment, positively interacting with microbiota, and the bioabsorption of standardized botanical extract constituents in their intact form. In addition, the Phytosome™ system is nanoparticle-free, and is considered safe and well tolerated. The present study aimed to evaluate, through an *in vitro* model of Normal Human Dermal Fibroblasts (NHDF) the antioxidant and anti-inflammatory capacity and the properties to counteract skin structural alteration exerted by a standardized *Centella asiatica* L. extract formulated with phospholipids (Centextra™) in comparison to unformulated *C. asiatica* L. dry extract.

## 2 Materials and methods

### 2.1 *Centella asiatica* L. extract

Assays were carried out with an innovative phospholipid (sunflower lecithin) carrier-based *C. asiatica* L. extract (Centextra™) characterized by a standardized content of total triterpens (as sum of asiatic acid, madecassic acid, asiaticoside, madecassicoside) ≥ 13.0% by HPLC and an unformulated dry *C. asiatica* L. extract (as sum of asiatic acid, madecassic acid, asiaticoside, madecassicoside) ≥ 45.0%. Solubilization was carried out according to the manufacturer’s instructions. Briefly, 100 mg of powder was suspended in 10 mL of ethanol 50% (v/v) in purified water, yielding a nominal concentration of 10 mg/mL and sonicated for 30 min at controlled room temperature (25 °C ± 2 °C). Tubes were kept closed to minimize solvent evaporation. Then samples were centrifuged at 10,000 × g for 10 min at 22 °C to remove insoluble material. Supernatants were collected and filtered through 0.22 µm PTFE filters under aseptic conditions. Samples were prepared fresh weekly, protected from light, and stored at 4 °C until use. Concentrations used in subsequent assays were expressed as extract dry weight equivalents and were not normalized to the content of individual triterpenes or total triterpene fraction.

### 2.2 NHDF cell culture

The study was conducted on Normal Human Dermal Fibroblasts (NHDF) from a single healthy donor (Promocell). Cells were grown in 75 cm^2^ cell culture flasks and maintained in culture with Fibroblast Growth Medium 2 (FGM2, Promocell) containing SupplementPack FGM2 (Promocell) and 1% Penicillin/Streptomycin (Life Technologies) to allow the best growth conditions. Cells were maintained in incubator at temperature of 37 °C in humidified atmosphere of 5% CO_2_ until confluence was reached. They were then propagated, by detachment with Trypsin/EDTA (Life Technologies), diluting them 1:6 or 1:9 for once a week.

### 2.3 Alamar blue assay (cells vitality test)

The Alamar Blue assay is designed to quantitatively measure cell viability and the toxic activity of different stimuli. Resazurin is a cell-permeable redox indicator that can be used to monitor the number of viable cells. Metabolically active viable cells reduce resazurin to resorufin, a pink and fluorescent product (O’brien et al., 2000). For this assay, fibroblasts were cultured in 48-well plates (3 × 10^4^ cells/well) in Fibroblast Growth Medium 2 (FGM2) supplemented with the FGM2 SupplementPack at 37 °C and 5% CO_2_. After 48 h, cells were exposed to different concentrations of the products under investigation (1000–1 μg/mL). Concentrations are expressed as extract dry weight equivalents and were not normalized to triterpene content. Cells were then incubated for an additional 48 h and subsequently treated with an Alamar Blue reagent solution (Merck Group) at a final concentration of 0.15 mg/mL. After 3 h of incubation at 37 °C protected from light, fluorescence was measured using a plate reader with excitation at 560 nm and emission at 590 nm (Bonnier et al., 2015). Results were expressed as the percentage of viable cells relative to the control for each compound. A negative control containing only the solvent was included to verify that its use did not induce any change in cell viability, and an untreated control was used as a reference for data normalization and comparison. All experiments were performed in two independent biological replicates, each including three technical replicates per condition. Statistical analysis was performed using one-way analysis of variance (ANOVA), followed by Dunnett’s *post hoc* test for multiple comparisons to evaluate differences between experimental groups and the control group. Data are expressed as mean ± SD, and a p-value <0.05 was considered statistically significant.

### 2.4 ROS assay

Intracellular ROS levels were measured using 2′,7′-dichlorodihydrofluorescein diacetate (H2DCFDA, Life Technologies). H2DCFDA passively diffuses into cells and is deacetylated by intracellular esterases to form the non-fluorescent compound 2′,7′-dichlorofluorescein (DCFH), which is subsequently oxidized by ROS to the fluorescent product DCF, retained within the cells (Kim and Xue, 2020). Fibroblasts were seeded at a density of 4 × 10^3^ cells/well in a 96-well plate. Forty-eight hours after seeding, cell cultures were pre-treated and incubated for an additional 48 h with various concentrations of the tested extracts (100–10 μg/mL, expressed as dry extract weight). Cells were then exposed to a menadione solution (vitamin K, Merck) at a concentration of 50 μM for 30 min to induce oxidative stress. To perform the assay, the culture medium was removed and cells were washed three times with PBS at room temperature. DCFH-DA, diluted to a final concentration of 10 μM, was added to the cultures and incubated for 30 min at 37 °C protected from light. Fluorescence was measured using a fluorescence plate reader with excitation at 485 nm and emission at 530 nm. The experiments were performed in two independent biological replicates, each including three technical replicates per condition and expressed as a percentage relative to the control. Statistical analysis was performed using one-way analysis of variance (ANOVA), followed by Dunnett’s *post hoc* test for multiple comparisons to evaluate differences between experimental groups and the control group. Data are expressed as mean ± SD, and a p-value <0.05 was considered statistically significant.

### 2.5 IL-6 assay

The quantification of interleukin-6 (IL-6) was performed using a commercial ELISA kit (Human IL-6 Confirm® ELISA Kit, Sigma-Aldrich) according to the manufacturer’s instructions. Cell culture supernatants, collected after pre-treatment with different concentration of the extracts (100–50 μg/mL, expressed as dry extract weight) and subsequent exposure to LPS at a concentration of 10 μg/mL, were centrifuged at 10,000 rpm for 10 min at 4 °C to remove any cellular debris. Enzymatic activity was measured spectrophotometrically using a plate reader at 450–590 nm. IL-6 concentrations in the samples were calculated by interpolating absorbance values on the standard curve. The experiments were performed in two independent biological replicates, each including three technical replicates per condition, pooled prior to analysis to minimize intra-condition variability. Each pooled samples and standards were analysed in quadruplicate. Values were normalized to the total protein content measured for each sample. Statistical analysis was performed using one-way analysis of variance (ANOVA), followed by Dunnett’s *post hoc* test for multiple comparisons to evaluate differences between experimental groups and the control group. Data are expressed as mean ± SD, and a p-value <0.05 was considered statistically significant.

### 2.6 Scratch assay

The scratch assay is an *in vitro* test used to study cell migration. This assay evaluates the ability of cells to migrate following the mechanical action of a scratch created with a sterile pipette tip on a confluent cell monolayer (Liang et al., 2007). Fibroblasts were seeded at a density of 2 × 10^6^ cells/well in 12-well plates. After 24 h, a longitudinal scratch was made in the center of each well using a sterile pipette tip. The culture medium was removed, and the cells were washed once with PBS to remove non-adherent cells. Fibroblasts were then treated with the two extracts (100–50 μg/mL, expressed as dry extract weight), and cell migration was monitored using an optical microscope at different time points (T0, T3, T6, T9, T24). Data analysis was performed using ImageJ software. The scratch area at the different time points was measured manually, and the obtained values were normalized to the initial area (T0). Cell migration was expressed as the percentage of wound closure:

$$\%\text{ closure}=\left(A_{t0}\;‐\;A_{\text{tx}}\right)\;/\;A_{t0}\times 100$$

The experiments were performed in two independent biological replicates, each including three technical replicates per condition. Statistical analysis was performed using one-way analysis of variance (ANOVA), followed by Dunnett’s *post hoc* test for multiple comparisons to evaluate differences between experimental groups and the control group. Data are expressed as mean ± SD, and a p-value <0.05 was considered statistically significant.

### 2.7 Collagen assay kit

Collagen quantification was performed using a commercial kit (Collagen Assay Kit, Sigma-Aldrich) according to the manufacturer’s instructions. Cell lysates, collected after pre-treatment with the different extracts (100–50 μg/mL, expressed as dry extract weight) and exposure to LPS (10 μg/mL) as explained before and obtained following protein extraction with RIPA buffer (Invitrogen), were clarified by centrifugation (11,000 × g, 10 min, 4 °C), and total protein content was quantified using a BCA assay (Thermo Fisher Scientific). In the first step of the assay, collagen is enzymatically digested and subsequently reacts with a dye reagent to form a fluorescent complex. The fluorescence intensity of the product was measured spectrophotometrically at 375 nm excitation and 465 nm emission. Collagen concentration in the samples was calculated by interpolating fluorescence values on the standard curve. The experiments were performed in two independent biological replicates, each including three technical replicates per condition. Values were normalized to the total protein content measured for each sample. Results were expressed as the percentage of collagen released relative to the LPS-treated cells. Statistical analysis was performed using one-way analysis of variance (ANOVA), followed by Dunnett’s *post hoc* test for multiple comparisons to evaluate differences between experimental groups and the control group. Data are expressed as mean ± SD, and a p-value <0.05 was considered statistically significant.

### 2.8 Collagenase assay kit

MMP-1 quantification was performed using a commercial ELISA kit (Human MMP-1 ELISA kit, Sigma-Aldrich) according to the manufacturer’s instructions. Cell culture supernatants, collected after pre-treatment with the different extracts (100–50 μg/mL, expressed as dry extract weight) and exposure to LPS (10 μg/mL) as explained before, were centrifuged at 10,000 rpm for 10 min at 4 °C to remove cellular debris. Enzymatic activity was measured spectrophotometrically using a plate reader at 450 nm. MMP-1 concentrations in the samples were calculated by interpolating absorbance values on the standard curve. The experiments were performed in two independent biological replicates, each including three technical replicates per condition, pooled prior to analysis to minimize intra-condition variability. Each pooled samples and standards were analysed in quadruplicate. Values were normalized to the protein content measured for each sample. Results were expressed as the percentage of MMP-1 released relative to the LPS-treated cells. Statistical analysis was performed using one-way analysis of variance (ANOVA), followed by Dunnett’s *post hoc* test for multiple comparisons to evaluate differences between experimental groups and the control group. Data are expressed as mean ± SD, and a p-value <0.05 was considered statistically significant.

## 3 Results

### 3.1 Alamar blue assay (cells viability test)

**FIGURE 1:**
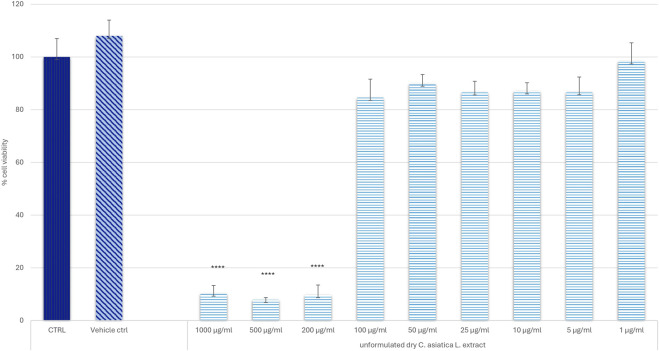
Effects of unformulated dry *Centella asiatica* L. extract on the viability of primary human fibroblasts after 48 h of exposure. Cell viability is expressed as percentage relative to untreated control cells. Data represent mean ± SD of six replicates (n = 6). Statistical significance was assessed by one-way ANOVA, followed by Dunnett’s *post hoc* test for multiple comparisons between experimental groups and the control group. * ≤0.05; ** ≤0.01; ***≤0.001; ****≤0.0001.

**FIGURE 2:**
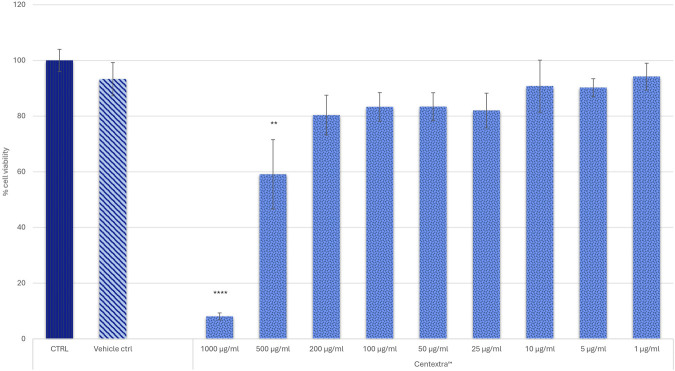
Effects of Centextra™ on the viability of primary human fibroblasts after 48 h of exposure. Cell viability is expressed as percentage relative to untreated control cells. Data represent mean ± SD of six replicates (n = 6). Statistical significance was assessed by one-way ANOVA, followed by Dunnett’s *post hoc* test for multiple comparisons between experimental groups and the control group. * ≤0.05; ** ≤0.01; *** ≤0.001; ****≤0.0001.

The effect of the extracts on cell viability was evaluated in primary human fibroblasts following 48 h of exposure to increasing concentrations (1000–1 μg/mL) of the two products, in order to define the non-cytotoxic concentration range suitable for subsequent functional assays. Cell viability was expressed as percentage in comparison to untreated control cells and, moreover, solvent control was included to discriminate extract-related effects from vehicle-induced toxicity. The results obtained are shown in Figures 1, 2.

Treatment with unformulated dry *C. asiatica* L. extract significantly reduced cell viability at the highest tested concentrations (1000–200 μg/mL), resulting in approximately 90% cell death compared with untreated control (p ≤ 0.0001; Figure 1). No statistically significant differences in cell viability were observed at lower concentrations.

Exposure to Centextra™ induced a reduction in cell viability only at 1000 and 500 μg/mL, corresponding to a 90% and 48% decrease relative to the control, respectively (p ≤ 0.0001 for 1000 μg/mL and p ≤ 0.01 for 500 μg/mL; Figure 2). No significant effects were detected at the other tested concentrations.

### 3.2 ROS assay

On the basis of the viability data, the antioxidant potential of the products was evaluated at non-cytotoxic concentrations by measuring intracellular ROS levels in fibroblasts subjected to menadione-induced oxidative stress. ROS levels were quantified relative to untreated control cells to evaluate the ability of each extract to counteract menadione-induced oxidative stress.

**FIGURE 3:**
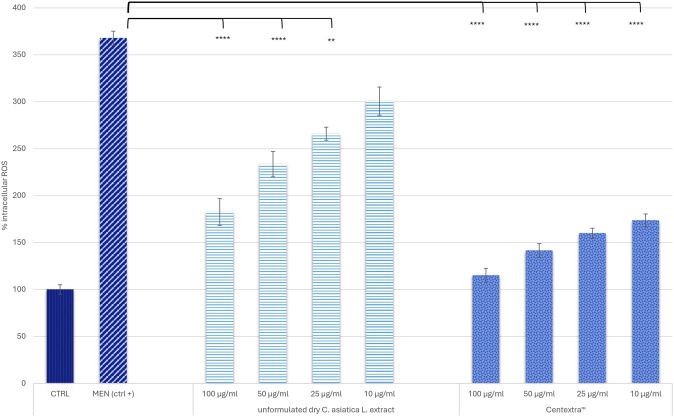
Effects of unformulated dry *Centella asiatica* L. extract and Centextra™ on intracellular ROS levels in primary human fibroblasts following menadione-induced oxidative stress. ROS levels are expressed as percentage relative to untreated control cells. Data represent mean ± SD of six replicates (n = 6). Statistical significance was assessed by one-way ANOVA, followed by Dunnett’s *post hoc* test for multiple comparisons between experimental groups and the menadione-treated group. * ≤0.05; ** ≤0.01; *** ≤0.001; ****≤0.0001.

As shown in Figure 3, Centextra™ significantly reduced intracellular ROS levels in a dose-dependent manner, restoring values comparable to those observed in untreated control cells. The reduction was statistically significant at all effective concentrations when compared with the menadione-treated condition (p ≤ 0.0001).

Unformulated dry *C. asiatica* L. extract also exhibited a dose-dependent antioxidant effect, leading to a significant decrease in intracellular ROS levels relative to menadione-treated cells (p ≤ 0.0001 for 100–50 μg/mL and p ≤ 0.01 for 25 μg/mL). However, the magnitude of the effect was overall less pronounced than that observed for Centextra™.

### 3.3 IL-6 assay

Since oxidative stress is closely associated with inflammatory signalling, the effects of the products on pro-inflammatory cytokine production were next evaluated by measuring IL-6 release in LPS-stimulated fibroblasts. IL-6 levels were expressed relative to the LPS-treated cells to assess the ability of the extracts to modulate pro-inflammatory cytokine production.

**FIGURE 4:**
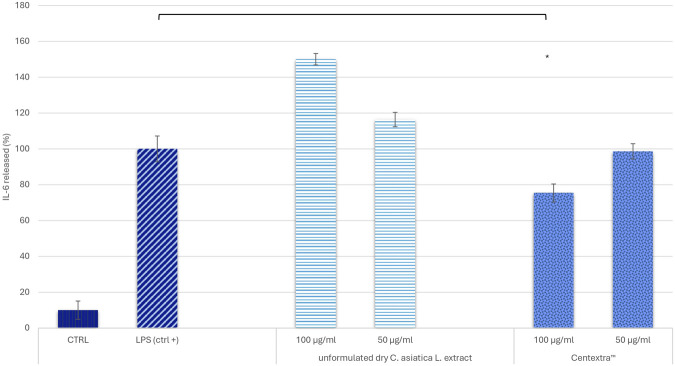
Effects of unformulated dry *Centella asiatica* L. extract and Centextra™ on IL-6 release in primary human fibroblasts following LPS-induced inflammatory stimulation. IL-6 levels were quantified by ELISA and are expressed as percentage relative to the LPS-treated cells. Data represent mean ± SD of four replicates (n = 4). Statistical significance was assessed by one-way ANOVA, followed by Dunnett’s *post hoc* test for multiple comparisons between experimental groups and LPS-treated control. * ≤0.05; ** ≤0.01; *** ≤0.001; ****≤0.0001.

In Figure 4, Centextra™ significantly reduced IL-6 levels only at the highest tested concentration (100 μg/mL) compared with the LPS-treated cells (p ≤ 0.05). No significant effects were observed at lower concentrations.

In contrast, unformulated dry *C. asiatica* L. extract did not induce a significant reduction in IL-6 levels at any of the tested concentrations, indicating a lack of measurable anti-inflammatory activity under the experimental conditions applied.

Notably, the modulatory effects on IL-6 level were observed predominantly at the highest tested concentration (100 μg/mL), indicating a concentration-dependent response with a relatively narrow effective range under the experimental conditions.

### 3.4 Scratch assay

Because fibroblast migration is a key component of tissue repair, the effects of the products on cell motility were assessed using an *in vitro* scratch assay. Cell migration was quantified as percentage of wound closure relative to the untreated control to assess the potential involvement of the extracts in processes related to tissue repair.

**FIGURE 5:**
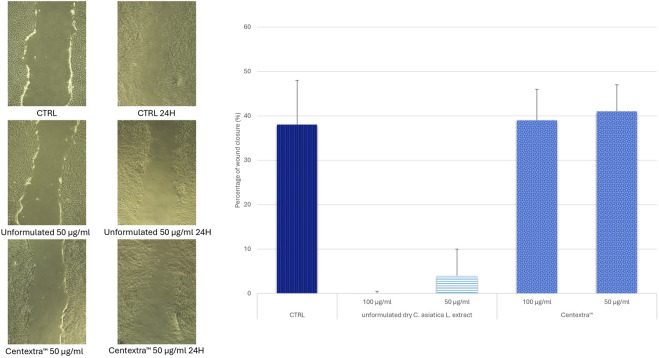
Effects of unformulated dry *Centella asiatica* L. extract and Centextra™ on fibroblast migration assessed by *in vitro* scratch assay. Cell migration is expressed as percentage of wound closure relative to untreated control cells. Data represent mean ± SD of six replicates (n = 6). Statistical significance was assessed by one-way ANOVA, followed by Dunnett’s *post hoc* test for multiple comparisons between experimental groups and the control group.

As shown in Figure 5, the two products exhibited markedly different effects on cell migration. Centextra™ extract resulted in wound closure comparable to control conditions (approximately 40% wound closure); however, no statistically significant differences were observed.

In contrast, unformulated dry *C. asiatica* L. extract did not induce any significant increase in wound closure at any tested concentrations, indicating a lack of measurable effect on fibroblast migratory activity under the experimental conditions applied.

### 3.5 Collagen assay kit

To determine whether the extracts could support matrix rebuilding under inflammatory conditions, collagen production was quantified in LPS-treated fibroblasts. Collagen levels were quantified relative to control conditions to determine the ability of the extracts to counteract LPS-induced impairment of collagen production.

**FIGURE 6:**
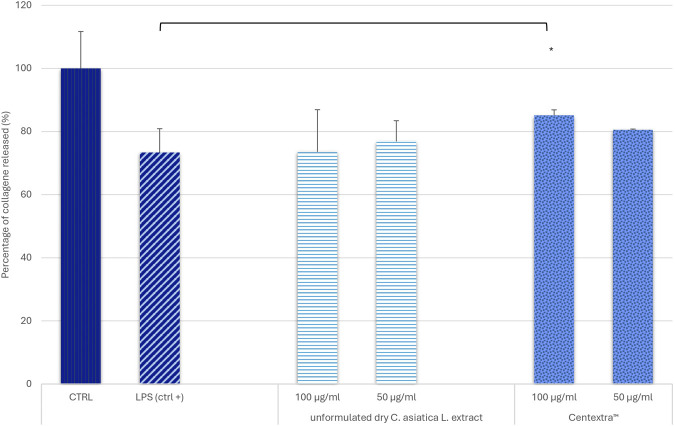
Effects of unformulated dry *Centella asiatica* L. extract and Centextra™ on collagen levels in primary human fibroblasts following LPS-induced inflammatory stimulation. Collagen content was quantified in cell lysates and is expressed as percentage relative to control conditions. Data represent mean ± SD of six replicates (n = 6). Statistical significance was assessed by one-way ANOVA, followed by Dunnett’s *post hoc* test for multiple comparisons between experimental groups and LPS-treated control. * ≤0.05; ** ≤0.01; *** ≤0.001; ****≤0.0001.

Centextra™ significantly restored collagen levels only at the highest tested concentration (100 μg/mL) compared with the LPS-treated cells (p ≤ 0.05, Figure 6). Collagen content was recovered to values closer to those observed under physiological (untreated) conditions.

Unformulated dry *C. asiatica* L. extract did not result in a significant increase in collagen levels at any of the tested concentrations, indicating the absence of a measurable effect on collagen synthesis under the experimental conditions applied.

Collagen restoration was likewise evident primarily at the highest concentration tested, supporting the presence of a narrow concentration window for measurable biological activity.

### 3.6 Collagenase assay kit

Since collagen homeostasis depends not only on synthesis but also on degradation, the effects of the compounds on matrix metalloproteinase-1 (MMP-1), a key collagen-degrading enzyme, were evaluated. MMP-1 levels were measured to assess the ability of the extracts to counteract LPS-induced extracellular matrix remodeling.

**FIGURE 7:**
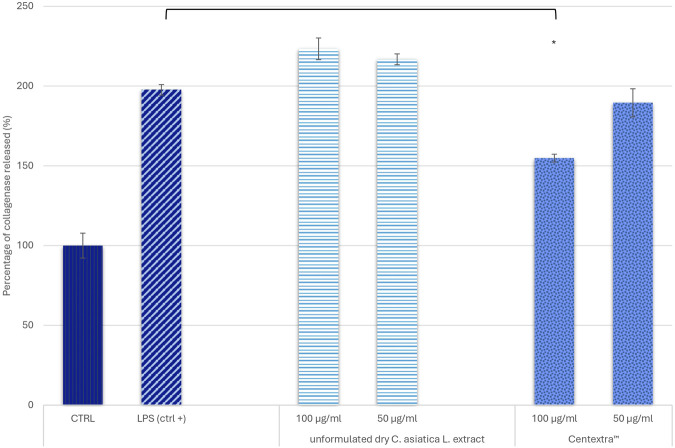
Effects of unformulated dry *Centella asiatica* L. extract and Centextra™ on MMP-1 release in primary human fibroblasts following LPS-induced inflammatory stimulation. MMP-1 levels were quantified by ELISA and are expressed as percentage relative to the LPS-treated cells. Data represent mean ± SD of four replicates (n = 4). Statistical significance was assessed by one-way ANOVA, followed by Dunnett’s *post hoc* test for multiple comparisons between experimental groups and LPS-treated control. * ≤0.05; ** ≤0.01; *** ≤0.001; ****≤0.0001.

As shown in Figure 7, exposure to LPS significantly increased MMP-1 release compared with untreated control. Centextra™ at the highest tested concentration (100 μg/mL) significantly reduced MMP-1 levels compared with the LPS-treated cells (p ≤ 0.05), whereas no significant effects were observed at lower concentrations.

Unformulated dry *C. asiatica* L. extract did not result in a significant reduction of MMP-1 release at any of the tested concentrations, indicating a lack of measurable inhibitory activity on collagenase production under the experimental conditions applied.

A similar pattern was observed for MMP-1, with significant modulation detected only at 100 μg/mL, suggesting that the effect is restricted to higher exposure levels.

## 4 Discussion

In this study, the beneficial effects of a standardized phospholipid carrier-based *C. asiatica* L. extract (Centextra™) were investigated comparing to an unformulated dry *C. asiatica* L. extract on skin health using an *in vitro* cell model. It is well established that inflammation and oxidative stress are increasingly recognized as central determinants of skin health and aging. ROS generated both endogenously and in response to environmental stressors disrupt redox homeostasis and directly damage cellular matrix (Pillai et al., 2005; Rinnerthaler et al., 2015). Oxidative stress also acts as a potent upstream driver of cutaneous inflammation by activating redox-sensitive signalling pathways leading to the sustained production of pro-inflammatory cytokines and matrix-degrading enzymes (Kammeyer and Luiten, 2015; Cole et al., 2018). This inflammatory–oxidative loop contributes to extracellular matrix breakdown, impaired barrier function, and dysregulated wound healing, ultimately accelerating both intrinsic and extrinsic skin aging (Quan et al., 2013; D’Orazio et al., 2013; Sucharitakul et. al, 2026). These findings highlight the relevance of interventions aimed at simultaneously reducing oxidative stress and inflammation as a rational strategy to preserve skin structure and function. Botanicals have long been recognized for their multifaceted bioactive properties, and their use in beauty-focused supplementation products continues to expand, leading to an increasing demand for natural and safe solutions to counteract the aging process signs. Furthermore, the increasing focus on healthy aging has reinforced the role by botanical ingredients capable of promoting long-term cellular resilience and structural maintenance of the skin, preserving cutaneous homeostatis (Ilma et al., 2025). Most recent research showed how aging organs are more interconnected than expected, which means the attention is shifting to provide natural preventive solutions able to exert pleiotropic effect (Jacquier et al., 2024). Moreover, botanical extracts with specific properties able to influence skin, hair, nails and with detoxifying mechanism of actions are called “Nutricosmetic” or “Cosmeceutical” and are growing very fast in a specific market size known as Beauty from Within (Dini and Laneri, 2019). Interest in beauty from within spans across age groups. While traditionally associated with mature consumers seeking to counteract signs of aging, younger individuals are increasingly adopting healthy routines that target skin health starting from the inside.

Botanicals, such as *Centella asiatica*, align well with this concept, as they offer bioactive compounds that may contribute to sustained dermal integrity, protection against environmental stressors, and modulation of age-related biological pathways (Handayani et al., 2025). Despite this evidence, the relevance of *Centella asiatica* triterpenes is constrained by well-documented absorption limitations at both intestinal and dermal levels. The main bioactive constituents (asiaticoside, madecassoside and related aglycones) exhibit poor aqueous solubility and unfavourable physicochemical properties, resulting in limited intestinal permeation and extensive presystemic metabolism after oral administration (James and Dubery, 2009; Anand et al., 2010; Handayani et al., 2025). These absorption constraints substantially limit systemic and local bioavailability, warranting caution when interpreting pharmacological effects in the absence of formulation strategies specifically designed to enhance tissue delivery.

For these reasons, the principal aim of the present study was focused on the evaluation of the efficacy of an innovative product of *Centella asiatica* extract formulated with Phytosome™ carrier system, in maintaining skin health and counteracting skin aging. The formulation proposed consisted in a phospholipid carrier that has been already used with other kind of compound and botanical agents, such as palmitoylethanolamide (Khan et al., 2026), *Boswellia serrata* extract (Riva et al., 2016), berberine (Petrangolini et al., 2021), curcumin (Cuomo et al., 2011), quercetin (Riva et al., 2019). The study was based on a comparison between two products: *Centella asiatica* phospholipids (Centextra™) and an unformulated Centella extract.

Data obtained indicated that the two products have been shown to modulate oxidative stress, relevant to skin homeostasis. The exhibited antioxidant activity obtained by reducing reactive oxygen species intracellularly in an *in vitro* skin model was likely attributed to triterpenoid constituents, including asiaticoside, madecassoside confirming as already reported in some papers (Park et al., 2017; Sun et al., 2020; Bandopadhyay et al., 2023). However, no direct mechanistic investigation was performed in the present study, and therefore any mechanistic interpretation remains speculative. Noteworthy, *Centella asiatica* phospholipids proved to be more efficient than the unformulated extract. This difference may reflect improved cellular interaction or availability of bioactive components, although no direct measurements of uptake or bioavailability were conducted.

In parallel, Centextra™ modulated inflammatory responses through the reduction of the pro-inflammatory cytokine IL-6 and the matrix metalloproteinases (MMP1) involved in extracellular matrix degradation in line with previous reports (Maquart et al., 1999; James and Dubery, 2009). However, in the absence of pathway-specific analyses, these results should be interpreted as functional outcomes rather than evidence of a defined mechanism of action.

Moreover, the functional assays related to tissue repair revealed that Centextra™ tended to increase wound closure in the scratch assay, but this effect did not significantly differ from untreated conditions; however, it showed greater wound closure compared with the unformulated extract, which failed to promote migration under the same conditions. In contrast, a significant modulation of collagen levels was observed under inflammatory stimulation. These findings suggest a potential influence on extracellular matrix turnover, although the effects were concentration-dependent and limited to specific experimental conditions.

In conclusion, this study demonstrated that a phospholipid-based Phytosome™ formulation of standardized *Centella asiatica* extract exhibited a more pronounced biological activity compared with the unformulated dry extract in an *in vitro* skin cell model across several analysis. These findings support the hypothesis that formulation is a critical determinant of the functional efficacy of *C. asiatica* triterpenes, whose intrinsic bioactivity is otherwise limited by poor solubility and restricted cellular availability. The phospholipid carrier system may be consistent with differences in physicochemical properties affecting cellular interaction, although this was not directly assessed, thereby translating the known pharmacological potential of *C. asiatica* into measurable protective and regenerative effects at the cellular level.

Some limitations should be acknowledged. Further studies are required to elucidate the molecular pathways in order to highlight the mechanism of action of this delivery strategy and the potential involvement of specific pathways as NF-κB, MAPK, Nrf2, as already documented (Hein et al., 2025). Moreover, the tested concentrations were expressed as total extract weight, without normalization to specific active constituents, limiting the interpretation of dose–response relationships at the molecular level. Furthermore, the use of NHDF commercially available derived from a single donor does not capture inter-individual biological variability but we decided to use primary cell culture (that are not easy to find) instead of immortalized cell lines because are more similar to the human condition. However, future studies including clinical studies are required to confirm the robustness and reproducibility of these findings, to confirm the translational relevance of these effects and to define their magnitude in the context of physiological skin aging and nutricosmetic applications.

In addition, detailed physicochemical characterization of the phospholipid formulation was not performed in the present study, limiting the ability to directly relate formulation properties to the observed biological effects.

Overall, the present results provide mostly scientific evidence that Phytosome™-based *C. asiatica* formulation may contribute to the modulation of oxidative stress- and inflammation-related parameters in an *in vitro* model.

## Data Availability

The datasets generated and/or analyzed during the current study are available from the corresponding author upon reasonable request. Requests should be directed to enrica.vella@indena.com.
